# Rapid and Accurate Detection of Urinary Pathogens by Mobile IMS-Based Electronic Nose: A Proof-of-Principle Study

**DOI:** 10.1371/journal.pone.0114279

**Published:** 2014-12-19

**Authors:** Antti Roine, Taavi Saviauk, Pekka Kumpulainen, Markus Karjalainen, Antti Tuokko, Janne Aittoniemi, Risto Vuento, Jukka Lekkala, Terho Lehtimäki, Teuvo L. Tammela, Niku K. J. Oksala

**Affiliations:** 1 School of Medicine, University of Tampere, Tampere, Finland; 2 Department of Automation Science and Engineering, Tampere University of Technology, Tampere, Finland; 3 Department of Clinical Microbiology, Fimlab Laboratories, Tampere, Finland; 4 Department of Clinical Chemistry, Fimlab Laboratories and University of Tampere, School of Medicine, Tampere, Finland; 5 Department of Surgery, School of Medicine, University of Tampere and Department of Urology, Tampere University Hospital, Tampere, Finland; 6 Department of Surgery, School of Medicine, University of Tampere and Department of Vascular Surgery, Tampere University Hospital, Tampere, Finland; Charité, Campus Benjamin Franklin, Germany

## Abstract

Urinary tract infection (UTI) is a common disease with significant morbidity and economic burden, accounting for a significant part of the workload in clinical microbiology laboratories. Current clinical chemisty point-of-care diagnostics rely on imperfect dipstick analysis which only provides indirect and insensitive evidence of urinary bacterial pathogens. An electronic nose (eNose) is a handheld device mimicking mammalian olfaction that potentially offers affordable and rapid analysis of samples without preparation at athmospheric pressure. In this study we demonstrate the applicability of ion mobility spectrometry (IMS) –based eNose to discriminate the most common UTI pathogens from gaseous headspace of culture plates rapidly and without sample preparation. We gathered a total of 101 culture samples containing four most common UTI bacteries: *E. coli*, *S. saprophyticus*, *E. faecalis*, *Klebsiella spp* and sterile culture plates. The samples were analyzed using ChemPro 100i device, consisting of IMS cell and six semiconductor sensors. Data analysis was conducted by linear discriminant analysis (LDA) and logistic regression (LR). The results were validated by leave-one-out and 5-fold cross validation analysis. In discrimination of sterile and bacterial samples sensitivity of 95% and specificity of 97% were achieved. The bacterial species were identified with sensitivity of 95% and specificity of 96% using eNose as compared to urine bacterial cultures. In conclusion: These findings strongly demonstrate the ability of our eNose to discriminate bacterial cultures and provides a proof of principle to use this method in urinanalysis of UTI.

## Introduction

Urinary tract infection (UTI) is a condition where pathogen enters urinary system either by ascending via urethra or via hematological route and causes an infection that can range from mild cystitis to life-threatening pyelonephritis [1]. UTI is one of the most common infections in humans. It is associated with considerable economical costs [2].

80% of all uncomplicated UTIs are caused by gastrointestinal bacteria *Escherichia coli*, approximately 15% are caused by *Staphylococcus saprophyticus*, a bacterium commonly present in female genital tract [3]. The remaining 5–10% are caused by *Klebsiella* species or *Enterococcus faecalis*, both originating from the gastrointestinal tract [3]. A complicated UTI occurs in a patient who has a predisposing factor such as diabetes or an abnormality in the urinary tract that lowers natural resistance and enables opportunistic infections. *E. coli* is the most common pathogen in this population as well, but the composition of other pathogens depends on the predisposing factor of the patient [3].

Physicians must distinguish UTI from other diseases that have a similar clinical presentation, some UTIs are asymptomatic or present with atypical signs and symptoms. The diagnosis of UTI relies on urinalysis and bacterial culture. Urinalysis is achieved either by on-site dipstick analysis, or by flow cytometry in a laboratory-setting [4]. Dipstick analysis detects urinary leucocytes, nitrate and blood, whose clinical implications are interpreted by a physician. Urinalysis offers more accurate analysis of aforementioned parameters and an estimation of bacterial content of the urine [4]. Dipstick analysis is effective in ruling out infection but positive results need confirmation by evaluation of pre-test probability or by culture [5]. Bacterial culture is the only method that offers definite knowledge of antibiotic susceptibility. The disadvantage of culture is that it typically takes a minimum of 24 hours to complete. Matrix-assisted laser desorption/ionization time-of-flight (MALDI-TOF) is the current state-of-the-art method that offers inexpensive identification of micro-organisms. It is able to identify the bacterial species in about 90% of the cases [6]. Its disadvantage is that high fixed costs make it only viable in high volume laboratories where its high throughput can be fully utilized.

An electronic nose (eNose) is a device that mimics the working principle of mammalian olfaction. It consists of an array of nonselective sensors, preprocessing electronics and a computer that interprets sensor signals by pattern detection [7]. It is designed for qualitative, not quantitative analysis. This enables eNose to analyze complex mixtures that would be unanalyzable with more sensitive methods such as gas chromatography mass spectrometry without extensive preparation [8]. These characteristics, handheld size, and relative affordability make eNose a tempting tool for detection of UTI.

The ability of eNose to discriminate cultured bacteria has been demonstrated in [9]–[15]. Preliminary studies of detection of UTI from cultured urine samples have also been published [16]–[18]. Studies have largely employed a single device that is bulky and not suitable for point-of-care use. Only a single study has employed a clearly handheld device in classification of eye infection pathogens [19].

To date, no studies employing a hand-held IMS-based eNose device have been published. We have recently demonstrated the ability of such device to discriminate malignant prostate cells from benign ones [20], as well as shown that this prostatic cancer can be differentiated from benign hyperplasia in clinical setting with urine gaseous headspace as sample material [21]. UTI is a significant confounding factor for diagnostic methods employing urine as sample material. UTI can also be comorbid or diagnostic option for urological malignancies or benign prostatic hyperplasia. In this study we demonstrate the ability of IMS-based eNose to discriminate cultured urinary pathogens with high sensitivity and specificity.

## Materials and Methods

### Bacterial cultures

Four most common UTI pathogens, *Escherichia col*i, *Staphylococcus saprophyticus*, *Klebsiella species* and *Enterococcus faecalis*, were included in the study. Pure culture samples were made to 92 mm×16 mm polystyrene Petri dishes (No. 82.1472, Sarstedt AG & Co., Nümbrecht, Germany) containing cysteine lactose electrolyte deficient (CLED) medium (LAB041, Lab M Limited, Lancashire, UK). Urine samples for pure cultures were acquired anonymously from patients in Tampere University Hospital collected and processed by Fimlab laboratories, the provider of laboratory services for Pirkanmaa Hospital District. No data on the clinical background of the patients was collected. The patients did not provide written consent, since according to the policy of Pirkanmaa Hospital District, it is not required for the use of bacterial isolates collected in routine practice and used for methodological development. This study was approved by the ethical committee of Tampere University Hospital (code: R10066). Identical culture plates without evidence of bacterial growth were used as sterile controls.

The sample contained a total of 101 samples including all four pathogens and sterile controls. Detailed description of samples presented in Table 1. All samples containing pathogens were marked with an individual identification number and were stored in the refrigerator between the measurements (2–8 °C).

**Table 1:** Description of bacterial culture samples.

Pathogen	Number of samples
*Escherichia coli*	20
*Staphylococcus saprophyticus*	19
*Klebsiella species*	20
*Enterococcus Faecalis*	21
CLED agar	21

The samples were analyzed in presented order.

Abbreviations: cysteine lactose electrolyte deficient.

### Device and measurement

A commercially available eNose (ChemPro 100i, Environics Inc., Mikkeli, Finland) was employed in this study. It is based on ion mobility spectrometry principle. The device consists of an ion mobility cell (IMCell) which features eight electrode strips that produce 16-channeled data. It contains a radiation source of Am-241 5.92 MHq and a heater element that maintains the cell at a constant temperature. In addition to IMCell, the device has six metal oxide-semiconductor sensors. Ambient air is utilized as carrier gas. The device is described in more detail by Utriainen et al. [22] Flow was set at 1.30 l/min and sensor temperature was on average 31.5°C. In order to reduce background noise, the air entering measurement chamber was filtered through activated carbon and was then fed to the sensor.

Every sample was soaked into water bath (36°C) and measured for approximately 15 minutes. Between the samples ambient air was measured for approximately 3 minutes and the empty Petri dish for approximately 4 minutes to avoid carryover from the previous sample. These time intervals were found sufficient in pre-study experiments. The switching time was registered to the spreadsheet program and compared afterwards to the log file made by the Windows-based software provided with the eNose device. The log file was then submitted for statistical analysis.

### Statistical analysis

Sample analysis was designed to mirror the clinical decision making. In the first phase, we created a classified that discriminates the samples either to sterile, or infected group. For the infected group we performed additional classification that aimed to identify the bacterial species in question.

Linear discriminant analysis (LDA) and logistic regression (LR) were employed to identify the classifier for discrimination of sterile and bacterial samples. Only LDA was employed in identification of bacterial species since LR is only suitable for discrimination into two classes. The generalization ability of the classifiers was tested by leave-one-out (LOOCV) and K-fold cross validation [23]. Principal component analysis is a linear projection of the high-dimensional data to a lower dimensional space retaining the maximum variance of the data [24]. Principal components are used to visualize the structure of the data.

## Results

In discrimination of sterile and infected samples, eNose achieved sensitivity of 95% and specificity of 97% using LR. Sensitivity of 90% and specificity of 96% were achieved with LDA. These results were validated with LOOCV. Confusion matrix of the classification is presented in Table 2.

**Table 2:** Classification results of bacteria vs sterile samples with LDA and LR.

		Predicted LDA		Predicted LR	
		Sterile	Bacteria	Sterile	Bacteria
True	Sterile	19	2	20	1
	Bacteria	3	77	2	78

Left columns shows the true class of the sample. Top rows identify used prediction model and how it classifies samples. Both methods achieve near perfect discrimination, LR demonstrating marginally better performance.

**Figure 1:**
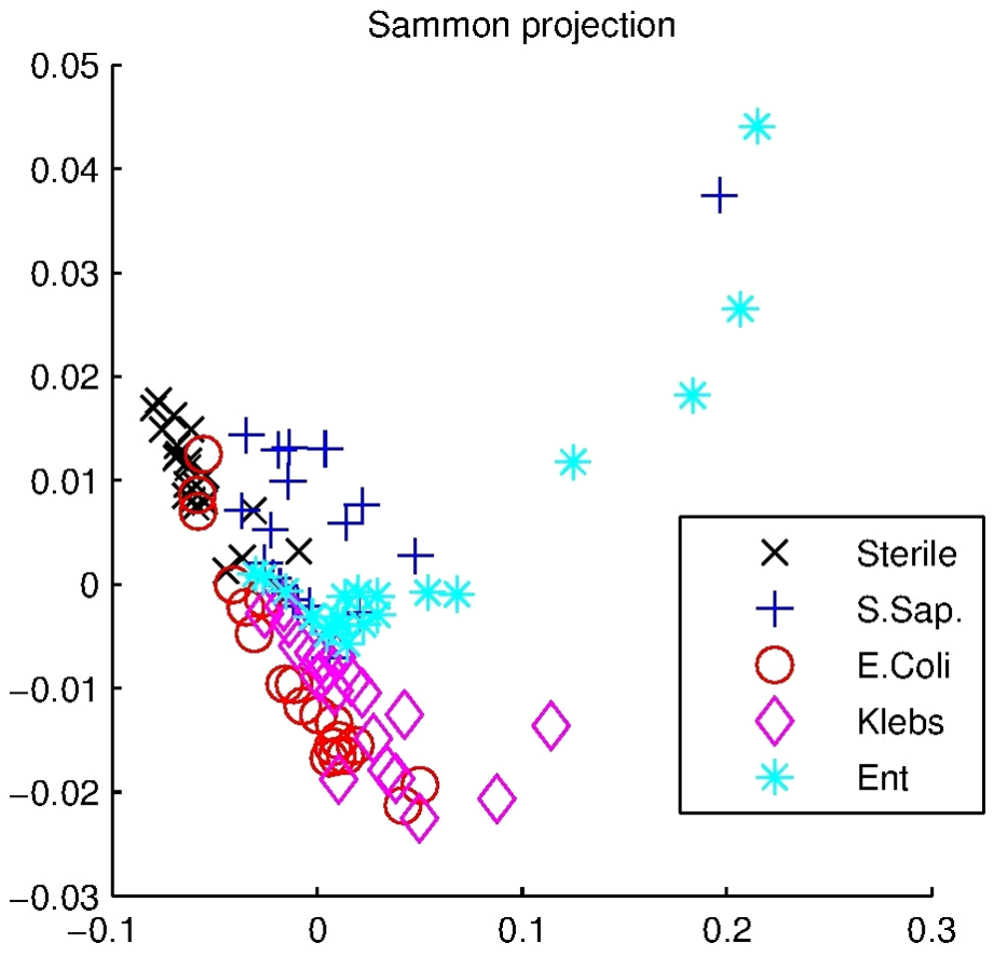
A plot visualizing the three principal components of the dataset used to classify samples into sterile or one of four bacterial species. Different species cluster in their own areas with some overlapping in 3-dimensional projections.

In classification of four different bacteria and sterile culture plate, LOOCV-validated sensitivity of 95% and specificity of 96% were achieved using LDA. One sterile sample was classified as *E. Coli*. All *Klebsiella spp* samples were all classified correctly. Three *E. coli* samples were classified as sterile. Discrimination of *S. saprophyticus* and *E. faecalis* was more challenging with seven *S.* saprophyticus samples classified as *E. faecalis* and five *E. faecalis* as *S. saprophyticus* samples. One *S. sapropyticus* and *E. faecalis* were misclassified as *Klebisiella spp*. This is in contrast with high discrimination of sterile, *E. coli* and *Klebsiella spp*. Classification results are presented in Table 3. Misclassification rates of LOOCV and five-fold cross validation are presented in Table 4. Monte Carlo simulation of 100 repetitions of five-fold cross validation provided mean misclassification rates of 18% for linear and 14% quadratic classifier. PCA plot in Fig. 1 visualizes how three principal components of the data present clusters with some overlapping.

**Table 3:** Identification of bacterial species and sterile samples.

		Predicted LDA				
		Sterile	*S.Saprophyticus*	*E.Coli*	*Klebsiella spp*	*E. faecalis*
True	Sterile	20	0	1	0	0
	*S.Saprophyticus*	0	11	0	1	7
	*E.Coli*	3	0	17	0	0
	*Klebsiella spp*	0	0	0	20	0
	*E. faecalis*	0	4	0	1	16

Left-hand colums identify true classification of the samples. Top row shows the discrimination by LDA. S. saprophyticus is most commonly misclassified and is often confused with E. faecalis. Overall discrimination is very high.

**Table 4:** Misclassification rates for classification of sterile vs bacteria and identification of bacterial species and sterile plate.

Classification		LOOCV (%)	Fivefold (%)
Bacteria vs sterile	LDA	4.9	5.0
	LR	3.0	4.0
Bacterial species and sterile plate identification	Sterile	0.0	0.0
	S. Sap.	36.8	21.0
	E. Coli	15.0	15.0
	Klebs	0.0	0.0
	Ent	28.6	23.8

Leave-one-out and K-fold cross validation are employed.

## Discussion

In this study we demonstrated the ability of affordable IMS-based handheld eNose to rapidly discriminate bacterial cultures with high accuracy at atmospheric pressure without sample preparation.

Previous works have focused on bacteria and yeasts in food industry [9], common bacterial and fungal human pathogens [10], [11], [13], [14], blood culture simulations [12]. The fact that every published study employed a different set of bacteria, makes direct comparison difficult. No studies focusing on sole discrimination of the most common urinary pathogen cultures have been published. Some studies have employed protocols of rapid incubation of urine, followed by analysis by eNose [15], [17], [18].

In all studies that focused solely on discrimination of bacterial cultures, complex sampling methods were used [9]–[11]; Bacterial cultures were sealed in a plastic bag where equilibrium of VOCs was allowed to form. The content of this bag was then fed to the sensor. In contrast, we employed a considerably simpler strategy where culture plate was simply connected to a plastic cover and measured immediately thus entirely omitting sample preparation. In the present study we used a relatively long measurement period of 15 minutes, but the fact that high discrimination can be achieved at 5 minutes already, suggests that the duration of measurement period can be significantly reduced. Based on our pilot experiments a minimum of four minutes of flushing with ambient air is required to avoid carryover from previous sample. In future we aim to improve the measurement cycle so that the duration can be reduced. This would directly translate to a higher throughput.

In point-of-care applications the mobility, rapidity and minimal cost of the device and analyses play a great role. A handheld device can be carried to patient's home for online analysis or used in medical wards without need to carry samples to centralized laboratory. A rugged, handheld device with minimal maintenance need would be suitable for use in developing countries. Commonly used conducting polymer sensor device Bloodhound BH114 is not suitable for mobile applications in its current form. Dutta et al used a handheld, battery powered Cyranose 320 device to discriminate pathogens responsible for eye infections [19]. The device relies solely on 32 conducting polymer sensors which are similar to six MOS-sensors in our device. They also achieved high overall discrimination of 96% with use of a combination of three simultaneous data clustering algorithms. By comparison, they only achieved a discrimination of 75% by PCA, while we achieved high sensitivity and specificity by using only LR. This shows that IMS is well capable of discriminating cultured bacteria responsible for UTI and may be even be better suited for the task than more traditional conducting polymer based eNose. Our device also has the advantage of long runtime and military-grade ruggedness which enable its use in difficult conditions.

Vernat-Rossi et al focused on discrimination of bacterium that are responsible for spoilage of dry meat products, with S. saprophytius as the only overlapping bacteria in our study [9]. They employed measurement chamber design similar to ours. By using LDA and LOOCV they achieved discrimination of 86% which is inferior compared to our present results. This may be due to different bacterial species but more likely explanation is relatively primitive eNose consisting of only six semiconductor sensors. Gibson achieved comparable discrimination but used a complex neural network approach [10]. Our sampling technique is powerful enough to produce good discrimination with relatively simple LDA and LR approaches in relatively small sample. It should be noted that current sample consists of highly standardized cultured samples with virtually no confounding factors. Schiffman et al. focused more on fungal growth and employed only a single culture of 10 different bacteria that were measured four times. Although they reached clear clustering of samples in LDA, LOOCV reduced performance significantly, indicating that discrimination model overfitted the small sample.

High classification achieved with *E. coli*, *Klebisella* spp and sterile culture plates was shadowed by poorer discrimination of *S. saprophyticus* and *E. faecalis*. This suggests that the two bacteria have similar smell prints. The two species were separated by other samples that could be classified with near-perfect performance, speaking against systematic confounder in some part of the measurements. No studies investigating volatile compounds released by *S. saprophyticus* exist, although *S. aureus* and *E. faecalis* have been extensively studied. They have fewer common volatile compounds than *Klebisella* spp and *E. coli* which were completely discriminated from each other [25]. *S. saprophyticus* seems to form two clusters, one overlapping with *E. faecalis* and another clearly separated from other bacteria. *S. saprophyticus* has two known subspecies: *saprophyticus* and *bowis*. An important distinction is that unlike *saprophyticus* subsp, *bowis* features nitrate reductase [26]. The enzyme catalyzes the production of nitrogen dioxide, a volatile compound that could potentially alter the smell print. *E. coli*, *Klebisella spp* and *E. faecalis* all have nitrate reductase, meaning that *S. saprophyticus* sp. *saprophyticus* is the only nitrate reductase –negative bacterium in this study, potentially explaining distinct clustering.

Limitations of this study are that bacteria were randomized within species but every species was carried out on separate batch. Complete randomization would rule out potential systemic factor that causes confusion of *E. faecalis* and *S. saprophyticus*. Another limitation is that standard culture in unable to discriminate subspecies of *S. saprophyticus*, which would clarify clustering of the bacteria. Another limitation is that we lack the knowledge of the clinical background of the patients. Although bacteria seem to grow at similar rates in diabetic and non-diabetic urine [27], it is possible that different environment in diabetic urine modulates the metabolism of bacteria and thus affects the smell of the cultures. We consider the effect of these metabolites minor, since the volume of urine (approximately 0.2 ml) compared the volume of Agar (approximately 20 ml) in culture plate results in significant dilution. Comorbid factors would be an important consideration in eNose analysis conducted straight from urine. UTI is more common in diabetic patients but the prevalence of *E. coli* is similar to non-diabetic population. *Klebisella* is 2–3 times more common in diabetic population, so it is likely that diabetic urine is enriched in these samples [3]. Smoking is associated with increased susceptibility to many diseases but, perhaps surprisingly, not to UTI [28].

With these promising results, the next step is to attempt discrimination of bacteria in urine samples. Aathithan et al. described an eNose device that could discriminate incubated urine samples as infected or uninfected after overnight incubation with sensitivity of 72.3% and specificity of 89.4% by use of PCA. No cross-validation was reported implicating that discrimination algorithm may suffer from overfitting the study sample and the performance may be significantly poorer in new sample set. The device used there was designed for laboratory setting and received an FDA approval. To authors' knowledge, the device is not currently available for commercial use. Kodogiannis et al used previously described Bloodhound device and by use of neural networks, managed to discriminate bacterial pathogens with a classification rate of 100%. Notably, this discrimination was achieved only after 5 hours of incubation. We aim to achieve similar discrimination with handheld device. The greatest challenge is that unlike cultured samples, urine samples are associated with high biological variance caused by kidney function, medications, age, sex and comorbid conditions such as inflammatory bowel disease which has recently been shown to modify the smellprint of urine due to changes in gut flora [29].

## Conclusions

A handheld, mobile eNose is able to rapidly, without sample preparation and at low cost to discriminate common urinary bacterial pathogens from culture plates. High discrimination is achieved both between sterile and bacterial plates as well as between different bacterial species. Our results warrant future trial attempting discrimination of urine samples. Future studies should focus on analysis of the effect of comorbidities such as diabetes and include a larger number of pathogens. Major attention should be paid on streamlining sampling and analysis to allow the analysis of larger samples.
